# P-1382. Utilization of Doxycycline Post-Exposure Prophylaxis at a Midwestern United States HIV/PrEP Clinic

**DOI:** 10.1093/ofid/ofae631.1558

**Published:** 2025-01-29

**Authors:** Gabriel Codling, Josh Havens, Elizabeth Lyden, Sara H Bares, Kimberly Scarsi, Shawnalyn Sunagawa

**Affiliations:** UNMC College of Pharmacy, Omaha, Nebraska; University of Nebraska Medical Center, Omaha, Nebraska; University of Nebraska Medical Center, Omaha, Nebraska; University of Nebraska Medical Center, Omaha, Nebraska; University of Nebraska Medical Center, Omaha, Nebraska; University of Nebraska Medical Center, Omaha, Nebraska

## Abstract

**Background:**

Limited real-world data exist regarding usage of doxycline post-exposure prophylaxis (doxyPEP). We evaluated appropriate doxyPEP prescribing and monitoring following implementation of a doxyPEP protocol at the University of Nebraska Medical Center (UNMC) Specialty Care Clinic.

**Figure:**
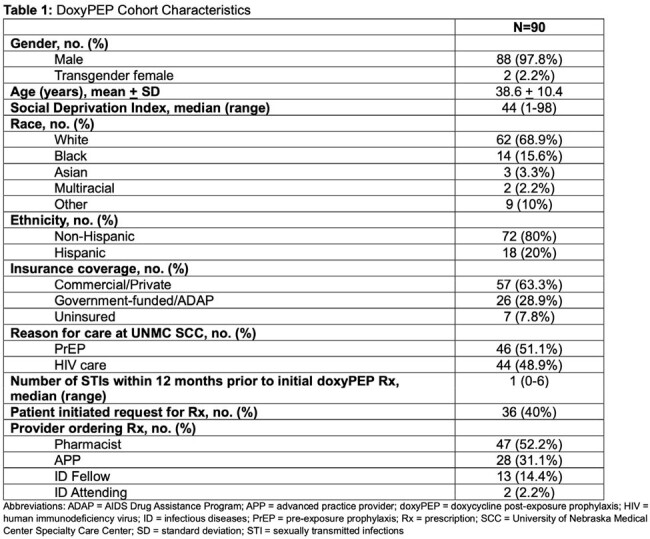

**Methods:**

We retrospectively evaluated patients receiving doxyPEP between 3/1/2023-2/29/2024. Patients receiving doxycycline for other indications were excluded. The primary outcome was adherence to the UNMC doxyPEP protocol. Secondary outcomes included total doxyPEP doses prescribed, sexually transmitted infection (STI) incidence in the doxyPEP cohort compared to 2023 UNMC STI incidence, and factors associated with protocol adherence or STIs while prescribed doxyPEP. We performed descriptive statistics for cohort characteristics and outcomes. Fisher’s exact test or student t-test was used to assess associations of patient characteristics with protocol adherence or STIs while on doxyPEP.

**Figure:**
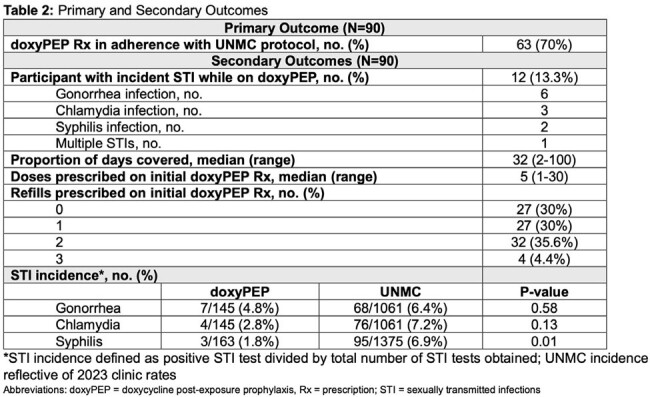

**Results:**

Characteristics of the cohort and prescriptions are listed in Table 1. Primary and secondary outcomes are reported in Tables 2 and 3. Of 90 patients receiving doxyPEP prescriptions, 30% were not prescribed in concordance with UNMC protocol; the most common reason was missed STI screenings (89%). There were 145 chlamydia/gonorrhea (CT/GC) and 163 syphilis screenings obtained from 90 patients; 13% of the patients on doxyPEP were diagnosed with an STI. Between patients prescribed doxyPEP and 2023 UNMC STI incidence, there was no difference in incidence of GC/CT (p > 0.05); however, syphilis incidence was lower (p=0.01) in patients on doxyPEP. There were no characteristics associated with doxyPEP protocol adherence. Age, reason for care, and number of STIs within 12 months of first doxyPEP prescription were significantly associated with an STI diagnosis while on doxyPEP.

**Figure:**
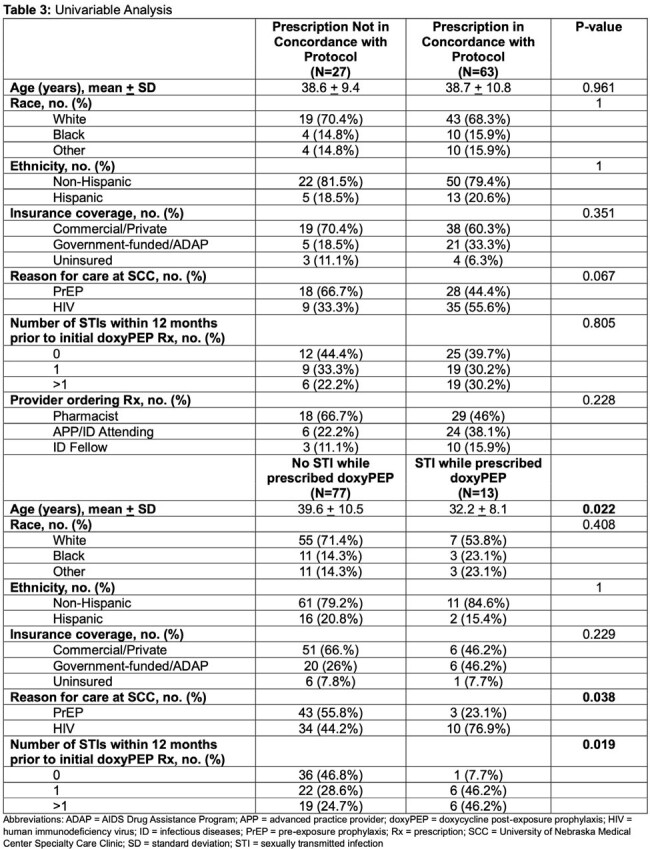

**Conclusion:**

Appropriate doxyPEP prescribing patterns occurred for 70% of prescriptions; missed STI screenings was the most common reason for protocol non-adherence. Patients with HIV and with > 1 STI in the past 12 months were more likely to be diagnosed with an STI while on doxyPEP. As utilization expands, it is necessary to ensure appropriate follow-up and monitoring.

**Disclosures:**

**Josh Havens, PharmD**, Gilead Sciences: Grant/Research Support|Medscape: Advisor/Consultant|ViiV Healthcare: Advisor/Consultant **Sara H. Bares, MD**, Gilead Sciences: Expert Testimony|Janssen: Grant/Research Support|ViiV Healthcare: Grant/Research Support **Kimberly Scarsi, PharmD**, Organon, Inc.: Grant/Research Support|Viiv Healthcare: Grant/Research Support

